# Mitochondrial Mechanisms of Apoptosis and Necroptosis in Liver Diseases

**DOI:** 10.1155/2021/8900122

**Published:** 2021-11-11

**Authors:** Qingfei Chu, Xinyu Gu, Qiuxian Zheng, Jing Wang, Haihong Zhu

**Affiliations:** State Key Laboratory for Diagnosis and Treatment of Infectious Diseases, National Clinical Research Center for Infectious Diseases, Collaborative Innovation Center for Diagnosis and Treatment of Infectious Diseases, The First Affiliated Hospital, College of Medicine, Zhejiang University, Hangzhou, Zhejiang 310003, China

## Abstract

In addition to playing a pivotal role in cellular energetics and biosynthesis, mitochondrial components are key operators in the regulation of cell death. In addition to apoptosis, necrosis is a highly relevant form of programmed liver cell death. Differential activation of specific forms of programmed cell death may not only affect the outcome of liver disease but may also provide new opportunities for therapeutic intervention. This review describes the role of mitochondria in cell death and the mechanism that leads to chronic liver hepatitis and liver cirrhosis. We focus on mitochondrial-driven apoptosis and current knowledge of necroptosis and discuss therapeutic strategies for targeting mitochondrial-mediated cell death in liver diseases.

## 1. Introduction

Cells undergo dynamic processes to maintain homeostasis, particularly in response to internal and external “stressors,” which include drugs, viruses, and molecules produced in metabolic disorders. The energy-driven control of internal cellular parameters to restore balance is known as the “stress response” [1]. Mitochondria are endocrine organelles that are key to the stress response [2], providing both the energy and the signals that enable direct stress adaptation [3]. Many stressors, such as toxic chemicals, mitochondrial DNA (mtDNA) mutations, reactive oxygen species (ROS) generation, and calcium signaling, directly target and interfere with mitochondrial function [4]. Other stressors may not directly target mitochondrial components, but their pathways often converge in mitochondria, reflecting their critical role of these organelles in stress adaptation. Mitochondrial fission and fusion are central in the cell stress adaptation process. Mitochondrial fusion helps alleviate stress by combining partially damaged mitochondria to restore organelle function. Fission is necessary for new mitochondria production, and it can also promote quality control by removing damaged mitochondria. Mitochondrial fission may be integral to programmed cell death, which is required for the removal of fatally damaged cells, which have exceeded their adaptive capacity. Undoubtedly, mitochondria play an essential role in apoptosis. Several studies have reported that members of the B cell lymphoma-2 (BCL-2) family drive mitochondrial outer membrane permeabilization (MOMP), resulting in the activation of apoptosis-inducing factor-dependent and caspase-dependent apoptotic cell death [5–7]. As a regulator of apoptosis, the Bcl-2 protein family includes both proapoptotic members and prosurvival members. Many studies have suggested ideas for further understanding BCL-2 family proteins, including their roles in controlling apoptosis.

In contrast to that of apoptosis, a well-defined form of cell death [8, 9], the model of necroptosis, regulated cell death with features distinct from those of apoptosis, is emerging [10]. Necroptosis can be activated by death receptor binding to their ligands, and these activated receptors associate with receptor-interacting protein kinase (RIPK) 1, which mediates RIPK3 activation, and mixed lineage kinase domain-like protein (MLKL) to form the necrosome [11, 12]. The necrosome, a signaling complex consisting of all three proteins, can alter plasma membrane permeabilization, leading to cell death. RIPK3 enhances aerobic respiration processes by mitochondria and increases the production of ROS. Mitochondrial respiration can in turn accelerate necrosome assembly [5]. However, research has shown that mitochondrial-deficient cells are resistant to mitochondrial-mediated apoptosis, although the death of these cells is activated by the tumor necrosis factor (TNF) signaling pathway [13]. Although ROS production relies on mitochondria, TNF-induced necroptosis is inhibited in mitochondrial-deficient cells, suggesting that the production of mitochondrial ROS accompanies, but is not necessary for RIPK3-dependent necroptosis [13].

In summary, both apoptotic and necroptotic cell death converge in mitochondria. However, more regulatory cell death mechanisms have been recently reported, including ferroptosis, pyroptosis, autophagy-dependent cell death (ADCD), mitochondrial permeability transition pore- (MPTP-) mediated necrosis, parthanatos, NETosis, and others [14]. Mitochondria may play essential functions in these alternative types of programmed cell death [15–17], but their molecular mechanisms are thus far poorly defined and, therefore, remain obscure. In this review, we describe the dynamic behavior (fusion and fission) of mitochondria in response to stress and mitochondrial-mediated mechanisms of cell death. Using this information, we discuss strategies for the treatment of chronic liver diseases, liver fibrosis, and hepatocellular carcinoma (HCC).

## 2. Mitochondrial Quality Control

Due to their crucial role in energy production, mitochondria are exposed to elevated levels of ROS and are thus especially sensitive to mtDNA mutations, and protein and lipid damage [18]. Impairment of mitochondrial function leads to high-level oxidant production, causing further mitochondrial damage, and the formation of a “vicious cycle.” Although the ROS generation that accompanies defects in oxidative phosphorylation or calcium overload contributes to cell death, ROS generation may be a downstream consequence of defects that primarily initiate mitochondrial dysfunction, and these functional defects are the core factors that determine cell survival or death.

**Figure 1:**
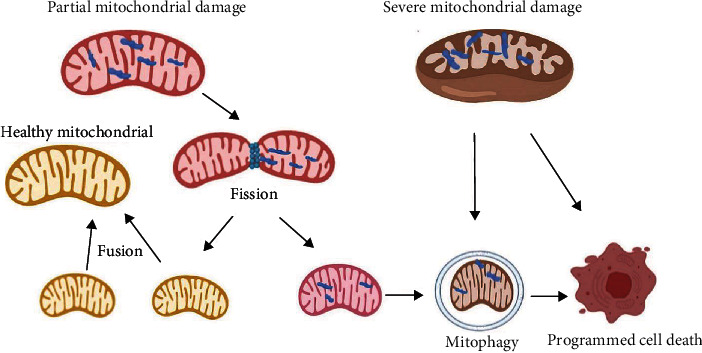
Role of mitochondrial damage in programmed cell death and preventive function of fusion, fission, and mitophagy. A partially altered mitochondrion (with blue blot) undergoes mitochondrial fission, leading to impaired mitochondria and a healthy small partner (without blue blot). The impaired components of mitochondria are eliminated through mitophagy. The two small healthy mitochondria generated undergo mitochondrial fusion to attenuate mitochondrial stress and restore mitochondrial function. However, when damage accumulates, a severely damaged whole mitochondrion is removed by mitophagy, and when the damage reaches a critical level, mitochondria will initiate programmed cell death.

Mitochondria remain in a stable state by the number of mitochondria and their form and/or function changing to meet the high demands of cellular bioenergetics and stress response pathways. In mammalian cells, damaged mitochondria are specifically identified and removed via autophagy (mitophagy) in a highly context-dependent manner that spares healthy organelle process known as “mitochondrial quality control” [19–21]. New mitochondria can be synthesized, and a healthy and functional mitochondrial population is thus maintained. The removal of dysfunctional mitochondria by autophagy has been shown by studies that also reported dysfunctional mitochondria and accumulation of swollen mitochondria in hepatocytes of mice that lack the essential autophagy genes AMPK and ULKE and, more recently, in mice that lack Atg7 [22, 23]. It has also been reported that damaged mitochondria induce MPTP opening, causing membrane depolarization that leads to selective mitochondrial engulfment by autophagosomes in hepatocytes [19]. Fusion and fission dynamics dilute and segregate damaged mitochondria [24]. Specifically, partially damaged mitochondria are segregated by dynamin-related protein 1- (DRP1-) mediated fission and then removed through mitophagy [25]. Mitochondria also maintain their intrinsic functions through fusion. A strict quality control system is essential. When mitochondrial damage reaches a critical threshold (in conditions of high cellular stress), the processes of mitochondrial fission, fusion, and mitophagy can fail to restore homeostasis, leading to disorders caused by high calcium levels, energy loss, and oxidative damage, ultimately inducing programmed cell death [24] (Figure 1).

## 3. Mitochondrial Regulation of Cell Death

### 3.1. Apoptosis

Mitochondrial dynamics and mitophagy, in several scenarios, contribute to stress adaptation that often prevents cell death. It might seem paradoxical, therefore, that mitochondria are important to the mechanisms that activate pathways of programmed cell death, such as apoptosis. Over the decades since apoptosis was first identified, many studies have explored the essential function of mitochondria in programmed cell death pathways.

**Figure 2:**
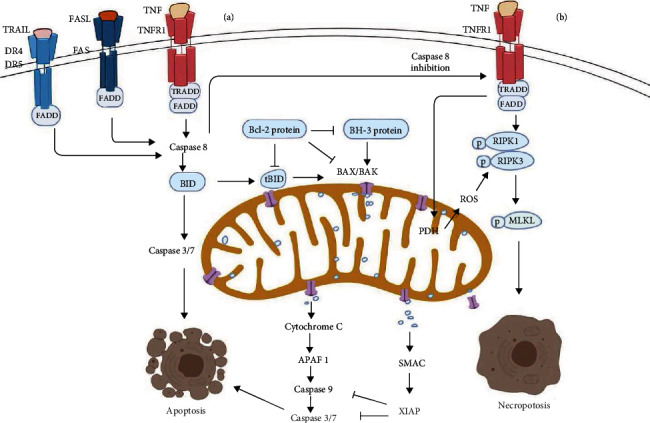
Mechanisms of mitochondrial regulation of apoptosis and necroptosis. Apoptosis proceeds mainly through the extrinsic and the intrinsic pathway. The TNFR superfamily (e.g., FAS, TNFR1, or DR4/DR5) is activated and interacts with death receptor ligands, FASL, TNF, or TRAIL. For example, TNF binds to and activates TNFR, recruiting the adaptor molecule FADD. Together, these proteins activate caspase 8, which cleaves and stimulates downstream caspase 3 and 7, which target hundreds of cellular components leading to rapid cell apoptosis. The pathway of mitochondrial-dependent apoptosis is instigated by intrinsic stimuli (including oxidative damage, DNA damage, growth factor depletion, or Ca^2+^ overload), which cause activation of BH3-only proteins. BH3-only proteins activate the effector proapoptotic proteins BAX and BAK, resulting in MOMP. Then, MOMP leads to the release of mitochondrial intermembrane space proteins, including cytochrome c. Cytochrome c interacts with APAF1, generating a special set of proteins called the apoptosome. The apoptosome interacts with caspase 9, which stimulates caspases 3 and 7. MOMP contributes to the release of cytochrome and SMAC. SMAC blocks the E3 ubiquitin-protein ligase XIAP (an inhibitor of caspases 3/7), promoting apoptosis. (b) After death receptor activation, the function of caspase 8 determines whether the cell undergoes apoptosis or necroptosis. If caspase 8 activation is inhibited, TNF signaling induces the activation of RIPK1 and RIPK3. RIPK3 phosphorylates and binds to MLKL causing the generation of necrosomes. The necrosome can be shuttled to the mitochondrial membrane and cause mitochondrial membrane permeabilization via a different mechanism of apoptosis. The necrosome also activates the mitochondrial pyruvate dehydrogenase (PDH) complex, enhancing aerobic respiration and ROS generation. In turn, ROS can increase the activation of RIPK3 and promote necrosome assembly.

There are two main types of apoptotic pathways: the extrinsic pathway initiated by external stimuli and the intrinsic (mitochondrial) pathway (a focus of this review) initiated in response to internal stimuli. Both types of apoptosis involve the action of caspases [26]. Caspases constitute a family of important cysteine proteases that are best known for cleaving hundreds of different proteins during apoptosis [27]. Since caspases themselves are cleaved during apoptosis, both apoptotic pathways are considered irreversible. Extrinsic apoptosis is activated by the TNF receptor (TNFR), apoptosis-independent surface antigen FAS, TNF-related apoptosis-inducing ligand (TRAIL) receptor death receptor (DR) 4, or DR5, binding with their respective ligand, leading to the recruitment of an adaptor molecule (FAS-associated death domain (FADD)) and activation of caspase 8. Then, activated caspase 8 cleaves and activates downstream caspases 7 and 3, which target hundreds of cellular components, rapidly leading to apoptotic cell death (Figure 2). To date, extrinsic apoptosis has not been linked to mitochondria.

Intrinsic apoptosis or the mitochondrial pathway of apoptosis is initiated by various intracellular disturbances (e.g., oxidative stress, DNA damage, calcium regulation imbalance, and endoplasmic reticulum stress) [28]. In the mitochondrial pathway, MOMP contributes to the release of soluble proteins, such as those in the mitochondrial intermembrane space. In addition to the aforementioned proteins, cytochrome c, a fundamental component of the mitochondrial electron transport chain, interacts with apoptotic peptidase activating factor 1 (APAF1), forming a complex called the apoptosome [29]. The apoptosome then binds to and activates caspase 9, which in turn cleaves and initiates executioner caspase 3 and 7. Simultaneously, MOMP leads to the release of SMAC and OMI, which block the caspase inhibitor XIAP, thereby facilitating apoptosis. Similar to the extrinsic pathway, the intrinsic apoptotic pathway involves the activation of caspase 8, which mediates the cleavage of BID (a BCL-2 family member). The cleavage product of BID, also known as truncated BID (tBID), is a mitochondrion-permeabilizing peptide [30, 31]. tBID triggers Bax (recruited from the cytosol) and Bak (located in the outer mitochondrial membrane), inducing MOMP and apoptosis (Figure 2). A recent study revealed an inhibitor of mitochondrial apoptosis called compound A that can inhibit Bax activation [32]. Importantly, MOMP results in massive water entry into the mitochondrial matrix, which causes mitochondrial swelling and expansion of the inner membrane, eliminating its cristae. MOMP, regulated by the Bcl-2 family [33], is considered a critical checkpoint in apoptosis, marking the point in the pathway where a cell is committed to death. In line with this notion, a study revealed that hepatocellular apoptosis is inhibited in Bid^−/−^ mice [34]. The proposed mechanism of MOMP induction by proapoptotic Bax and Bak mainly centres on their oligomerization to create lipidic pores in the outer membrane of a mitochondrion. During apoptosis, BH3-only proteins (a proapoptotic BCL-2 family protein, such as Bid) target and bind to Bax and Bak, inducing their oligomerization into higher-order multimers, which form lipidic pores, resulting in MOMP [5].

### 3.2. Necroptosis

Different types of cell death have distinct morphological and biochemical features. In the context of external injury, a cell can reach a certain state of energy depletion and lose basic functions, resulting in necrosis, through which cell membranes and organelles undergo uncontrolled disruption. Necrosis is an unregulated cell death and often involves the release of various proinflammatory factors, resulting in obvious inflammation and tissue damage [35]. Apoptosis is characterized by cytoplasmic and nuclear condensation (causing cell shrinkage) and cell membrane blebbing, but in contrast necrosis, in apoptosis, the membrane is not disrupted and no cellular components leak from the cells, and therefore, apoptosis fails to stimulate an inflammatory response [36]. Necroptosis is a newly discovered form of programmed necrosis that has features of both necrosis and apoptosis; for example, necroptosis is a form of programmed cell death and causes inflammation and tissue damage [37, 38].

Recently discovered necroptosis follows a programmed cell death pathway induced by TNF-*α* receptor activation. Activation of TNFR subsequently activates RIPK1 and RIPK3 [39]. In liver diseases, including drug-induced liver injury, autoimmune hepatitis, nonalcoholic steatohepatitis, and viral hepatitis, Toll-like receptor (TLR) signaling can activate necroptosis, but the TNF pathway is the best characterized to date. In a simplified model, when caspase 8 is inhibited, the activation of TNFR leads to the activation of RIPK1 and RIPK3, inducing the generation of necrosomes. RIPK3 in necrosome stimulates oligomerization of MLKL, which mediates necroptosis by forming cell-lytic plasma membrane pores [38, 40]. Concomitantly, damage-associated molecular patterns (DAMPs) and inflammatory cytokines are released by the cell.

What is the role of mitochondria in necroptosis? Studies have shown that in TNF-*α*-induced necroptosis, the interaction of RIP3 with MLKL induces translocation of the RIPK1/RIPK3/MLKL necrosome complex to the mitochondrial membrane where RIPK3 has been shown to activate a number of targets through phosphorylation, with more targets increasingly being discovered. One target is phosphoglycerate mutase 5 (PGAM5), which can recruit mitochondrial DRP1, leading to mitochondrial fragmentation [41]. In a feedforward cycle, RIPK3 kinase stimulates the pyruvate dehydrogenase complex, enhancing flux through oxidative respiration and generation of more ROS [42]. ROS levels can be crucial factors in the process of necroptosis and enhanced mitochondrial function because they increase the tendency of cells to undergo necroptosis. In addition, some evidence links RIPK3 to metabolic enzymes in the mitochondrial matrix, such as glutamine synthetase and glutamate dehydrogenase, promoting bioenergetic pathways and ROS production, leading to necroptosis. Many reports have suggested that necroptosis requires mitochondrial ROS generation via a mechanism dependent on mitochondrial permeability protein and cyclophilin D but independent of Bax or Bak [43]. However, it has also been shown that cells depleted of mitochondria through forced mitophagy can undergo necroptosis, implying that mitochondria or mitochondrial metabolism are not essential for necroptosis [13].

Further experimental research is needed to determine whether the mitochondrial ROS pathway is cell-specific or universal to all cells as an essential component of programmed necrosis (necroptosis) [44]. Although cell death was initially thought to be the result of inflammation, recent studies increasingly suggest that cell death may trigger or amplify the inflammatory response [45].

## 4. Mitochondrial-Mediated Cell Death in Liver Diseases

Damage to mitochondria and hepatocytes can be affected by a wide variety of factors, including viral infection, inflammatory responses, autoimmunity, toxic drugs, and ethanol. It is now clear that mitochondria are crucially important organelles in the cellular response to cell stress, and their functional state has a considerable impact on cell health. In this section, we describe the mechanisms involved in mitochondrial changes and damage in the context of chronic liver disease, cirrhosis, and HCC.

### 4.1. Mitochondrial and Chronic Hepatitis

**Figure 3:**
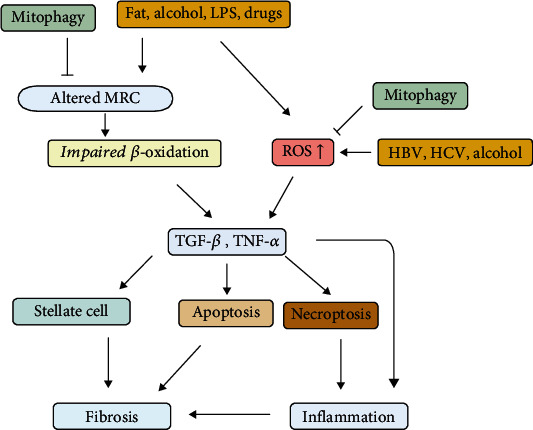
Mechanisms of mitochondrial involvement in hepatocyte death and fibrosis in chronic liver diseases. ROS can weaken *β*-oxidation, oxidize fat deposits, and prevent electrons from flowing along with the MRC. Disease factors such as chronic viral infection can contribute to increasing ROS levels and accelerated lipid peroxidation. Lipid peroxidation products increase the hepatic production of TGF-*β*, which activates hepatic stellate cells, leading to fibrosis. ROS also increase the synthesis of TNF and several other cytokines in the liver, which can cause apoptosis and necroptosis. Necroptosis induces mitochondrial DAMPS, which contribute to the production of inflammatory cytokines that promote liver inflammation.

Mitochondria not only provide energy for cells but are crucial to the dynamic maintenance of internal cellular parameters to ensure homeostasis in response to internal and external stresses on the cell. Mitochondrial fusion, fission, and mitophagy all play a role in maintaining this balance. Studies have revealed that mitochondria are involved in the development of hepatocyte steatosis, which is ubiquitous in chronic hepatitis, including that in a range of diseases from alcoholic liver disease (ALD) and nonalcoholic fatty liver disease and drug-associated liver diseases, as well as hepatitis B and C. Ultrastructural mitochondrial changes together with unbalanced mitochondrial-regulated cell dynamics can be found in patients with chronic liver disease [46, 47]. Lee et al. [48] pointed out that mitochondrial double-stranded RNA (dsRNA) in exosomes promotes the production of interleukin-17 in alcoholic liver injury through Toll-like receptor 3. These results suggest that mtdsRNA and TLR3 can be possible therapeutic targets to improve the treatment of ALD. Intriguingly, some studies revealed that, in the early stages of chronic liver disease, the liver can compensate through adaptation of mitochondria and mitophagy to reduce the effect of these diseases on the balance of cell dynamics; however, this compensatory effect is subsequently lost as disease severity increases. Mitophagy appears to be the primary response following the accumulation of dysfunctional mitochondria [49]. Chronic disease stimuli (ischaemia/reperfusion, drugs, alcohol, a high-fat level, lipopolysaccharides (LPSs), and diet-induced liver injury) lead to mitochondrial ROS generation and result in mtDNA damage [47, 50, 51]. This increased ROS generation can be an indirect result of detrimental effects to specific *β*-oxidation enzymes (or their cofactors) or a result of direct interference with a molecule in the mitochondrial respiratory chain (MRC). Some research has implicated the MRC and oxidative stress in the occurrence and progression of chronic hepatitis B virus (HBV) or hepatitis C virus (HCV) infection [49, 52, 53] (Figure 3).

ROS and impaired *β*-oxidation increase the synthesis of cytokines, such as TGF-*β* and TNF-*α*; these cytokines, in turn, cause apoptosis and necroptosis of hepatocytes [54–56]. Hepatocytes with mitochondrial damage and that die release DAMPs of mitochondrial origin, which can greatly stimulate the immune response and the release of inflammatory cytokines, resulting in chronic liver inflammation [57–59] (Figure 3).

In conclusion, the effects of chronic liver disease stimuli on mitochondria, via disruption of hepatic lipid metabolism and oxidative stress, lead to cell death (apoptosis and necroptosis) and inflammatory responses, which together with immune responses, result in liver fibrogenesis and liver fibrosis [55, 60, 61]. In addition, the danger signals derived directly from hepatocyte mitochondria activate hepatic stellate cells and promote the progression of liver fibrosis [62].

### 4.2. Mitochondrial and Liver Cirrhosis

In liver cancer and chronic liver diseases with serious complications, the key pathological process is cirrhosis. Prominent features of liver cirrhosis, such as insufficient liver tissue perfusion, ischaemia, hypoxia, and endotoxaemia, can all be linked to mitochondrial dysfunction.

**Figure 4:**
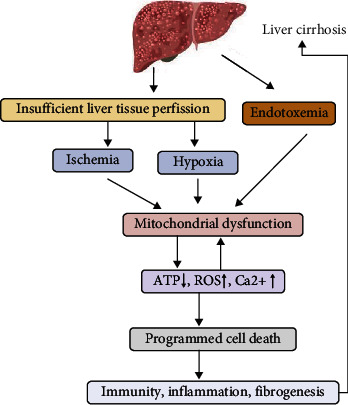
Mitochondrial dysfunction and liver cirrhosis. In liver cirrhosis, stimuli such as ischaemia, hypoxia, and endotoxaemia contribute to mitochondrial dysfunction. First, exposure of liver cells to a high level of endotoxin from the gastrointestinal tract inhibits enzyme dehydrogenase activity and blocks energy generation, and ROS production increases due to this interference of respiratory chain electron transfer. Second, unbalanced ROS levels lead to altered mitochondrial membrane permeability, induction of inflammatory signaling, and promoted activation of programmed cell death pathways. Additionally, ischaemia and hypoxia in the cirrhotic liver can cause cell membrane destruction, and Ca^2+^-ATPase activity at the cell membrane is inhibited. Ca^2+^ intake is often accompanied by depolarization of the mitochondrial membrane and H^+^ excretion. When the Ca^2+^ concentration in the mitochondria is excessive, inner membrane pores are opened. As a result, mitochondrial membrane permeability is increased. Mitochondria become dysfunctional, which induces programmed cell death, death-related inflammation, the immune response, fibrosis, and regeneration and further aggravates cirrhosis, thereby forming a vicious cycle.

Experiments on cirrhosis have proven that liver cells are frequently exposed to a high level of intestinal endotoxin. In these cells, mitochondria, which normally respond very dynamically to stressors to restore homeostasis, appear to respond weakly. The endotoxin binds to specific receptors to inhibit enzyme dehydrogenase activity in the oxidative respiration chain, blocking energy production and impairing the MRC, which not only affect antioxidant enzyme activity but also partially block the flow of electrons, reducing the production of oxygen and leading to the accumulation of superoxide anion production within respiratory complexes I and III [63]. The resulting imbalance of ROS can lead to further damage of OXPHOS proteins and mtDNA [64–66]. Thus, mitochondrial dysfunction is aggravated, and activation of programmed cell death pathways is promoted. Moreover, the ischaemia and hypoxia of liver tissue in cirrhosis cause MRC impairment and Ca^2+^ imbalance. Ca^2+^-ATPase activity at the membrane is inhibited by a large influx of extracellular Ca^2+^ accompanied by depolarization of the mitochondrial membrane and H^+^ excretion [67]. When the Ca^2+^ concentration in mitochondria is excessive, inner membrane pores are opened. As a result, mitochondrial membrane permeability is increased, leading to cell death (Figure 4).

In conclusion, during cirrhosis, mitochondria become dysfunctional, which further induces programmed cell death, death-related inflammation, immune response, and fibrosis, all of which further aggravate cirrhosis, forming a negative vicious cycle.

### 4.3. Mitochondria and HCC

We also briefly summarized the role of mitochondrial fission and fusion in the occurrence and development of HCC. Sun et al. [68] found that mitochondrial fission significantly promoted the reprogramming of adhesion dynamics and lamellipodia formation in HCC cells by activating the typical Ca^2+^/CaMK/ERK/FAK pathway. In addition, treatment with mitochondrial division inhibitor-1 can significantly reduce the calcium signal in HCC cells, which has a potential therapeutic effect on HCC metastasis *in vivo*. Mitochondrial fission can also promote tumor-associated macrophage infiltration and HCC progression by inducing mtDNA stress [69]. Li et al. [70] revealed that an increase in mitochondrial fusion changes metabolism and promotes the proliferation of HCC cells. Optic atrophy 1 (OPA1) and mitofusin 1/2 (MFN1/2) are at the core of the mitochondrial fusion process and are critical for the fusion of the inner and outer membranes, respectively [71]. Studies have shown that the downregulation of OPA1 or MFN1 inhibit the fusion process of HCC cell lines and cholangiocarcinoma (CCA) tumor organoids, inhibiting the growth of tumors in the body. However, studies have also revealed that MFN1 can inhibit the proliferation, invasion, and migration of HCC cells while promoting mitochondrial fusion. MFN1 inhibits liver cancer malignancies by driving the balance of mitochondrial dynamics from fission to fusion, which mediates the metabolic shift from aerobic glycolysis to oxidative phosphorylation [72]. These two outcomes do not seem to be contradictory, because mitochondrial dynamics are very complex, involving multiple links and multiple steps, and the impact on a disease depends on the type of disease and its specific background. Therefore, different observations in different studies are typical.

## 5. Therapies and Perspectives

Mitochondria play an essential role in distinct hepatocyte death, suggesting that efficient strategies to target mitochondrial-induced cell death pathways may hold particular promise for liver disease. A current drawback is that animal models used to study the mechanisms of chronic liver diseases and liver cirrhosis lack all the features of human disease. Some existing strategies have been discovered to target mitochondrial dysfunction in liver diseases, but these are likely to induce many nonspecific effects. It will be important to develop and test small molecules that target specific steps in mitochondrial-mediated cell death signaling pathways. For example, inhibitors of MOMP have been considered as cytoprotective agents [73]. Additionally, with respect to molecular targeting of cell death pathways, advances in gene manipulation technology may enable the correction of mutant genes important to mitochondrial-mediated pathways, thereby altering the course of certain liver disorders.

Targeting mitochondrial dynamics as a strategy for the treatment of mitochondrial disorders has received widespread attention [24]. Efforts have focused on enhancing mitophagy and controlling the balance between fusion and fission. For example, Mdivi-1, an inhibitor of DRP1, has been shown to be a potential treatment option for a variety of diseases [74, 75]. In addition, in chronic HBV or HCV infection, viruses direct mitochondrial mitophagy to evade immune surveillance, and strategies to activate mitophagy may effectively reduce the progression of liver injury involving mitochondrial damage and dysfunction. The correlation of plasma levels of DAMPs with mitochondrial-derived (for example, mtDNA) fibrosis, liver inflammation, or cirrhosis stage may be used to diagnose, monitor disease progression, or identify patients who are at risk.

## 6. Conclusions

In summary, improving our understanding of mitophagy and mitochondrial-driven cell death pathways may lead to a potential therapeutic target for liver diseases. However, much work needs to be done before safe and effective mitochondrial-driven cell death pathway inhibitors or drugs that improve mitochondrial function can be discovered. Progress toward these discoveries also depends on the successful translation of related research to humans.

## Abbreviations

mtDNA: Mitochondrial DNA
ROS: Reactive oxygen species
BCL-2: B cell lymphoma-2
MOMP: Mitochondrial outer membrane permeabilization
RIPK: Receptor-interacting protein kinase
MLKL: Mixed lineage kinase domain-like protein
TNF: Tumor necrosis factor
ADCD: Autophagy-dependent cell death
MPTP: Mitochondrial permeability transition pore
HCC: Hepatocellular carcinoma
DRP1: Dynamin-related protein 1
TNFR: TNF receptor
TRAIL: TNF-related apoptosis-inducing ligand
DR: Death receptor
FADD: FAS-associated death domain
APAF1: Apoptotic peptidase activating factor 1
TLR: Toll-like receptor
DAMPs: Damage-associated molecular patterns
PGAM5: Phosphoglycerate mutase 5
ALD: Alcoholic liver disease
dsRNA: Double-stranded RNA
LPSs: Lipopolysaccharides
MRC: Mitochondrial respiratory chain
HBV: Hepatitis B virus
HCV: Hepatitis C virus
OPA1: Optic atrophy 1
MFN1/2: Mitofusin 1/2
CCA: Cholangiocarcinoma.
